# Supplementary material for: Improved Skin Barrier Function Along with Hydration Benefits of Viola yedoensis Extract, Aesculin, and Schaftoside and LC-HRMS/MS Dereplication of Its Bio-Active Components
Source: Int J Mol Sci. 2024 Nov 27;25(23):12770. doi: 10.3390/ijms252312770 (PMC11641536; doi:10.3390/ijms252312770)
Supplement: Supplementary file 1 [file ijms-25-12770-s001.zip › ijms-3310864-supplementary.pdf]

Supplementary information

- **Figure S1.** Fragmentations of deprotonated luteolin by two competitive processes inducing the cleavage of: (a) the C2-C1′ linkage or (b) the C2-O bond.
- **Figure S2.** (a) charge delocalization comparison of deprotonated cichoriin [1-H]- and aesculin [2-H]-, (b) stepwise dehydrated glucose release from a particular cichoriin deprotomer through isomerization in a common ion/dipole complex prior to splitting by hydride transfer from the alkoxide site to the quinone aglycone moiety yielding the deprotonated esculetin and dehydrated hexose neutral as final state.
- **Figure S3.** Qualitative energy pathways of the stepwise dissociation of both the cichoriin [1-H]− and aesculin [2-H]− ions through the endothermic (i) and (i′) isomerization (see Figure 3) into a common IDp intermediate prior to the (ii) dissociation into a common product ion m/z 177 by release of dehydrated hexose. Transition state position of the (i′) pathway relative to that of (i) is difficult to be localized without quantum calculations.
- **Figure S4.** Proposed mechanisms of 3-hydroxy-3-methylglutaryl end group dissociations yielding (a) the consecutive losses of CO2 and (CO2 + C3H6O), (b) the complementary m/z 143 and m/z 371 ions via ion/dipole complex intermediate, and the m/z 143 competitive dissociations to form ions (c) m/z 99 and (d) m/z 125.
- **Figure S5.** Proposed stepwise mechanisms yielding m/z 249.1848 (C16H25O2) and m/z 191.1792 (C14H23) product ions of m/z 439.3567 (C30H47O2) via ion/dipole complex dissociations either directly or by internal proton transfer followed ion/dipole splitting, respectively.
- **Figure S6.** Proposed mechanisms of calenduloside F initiated by the specific C-O ester bond cleavage at the D/E ring junction preferred to that of the O-C ether bond at the A ring of the aglycone. This requires stepwise processes based on the isomerization of a deprotonated mole-cule into an ion/dipole complex to form product ions: either directly at m/z 631 (by loss of dehy-drated hexose), or by long-distance proton transfer from the D/E ring junction to the A ring, enabling release of dehydrated glucuronic acid by cleavage of the C-O ether bond (m/z 455). This loss competes with the release of H2O and CO2 to form m/z 613 and m/z 560, respectively.
- **Figure S7.** HPLC chromatogram of hydroalcoholic extract and fractions of *Viola yedoensis* by flash chromatography.
- **Figure S8.** Hygrometric probe.
- **Table S9.** Mobile phase gradient used in flash chromatography.

C1 - Internal use

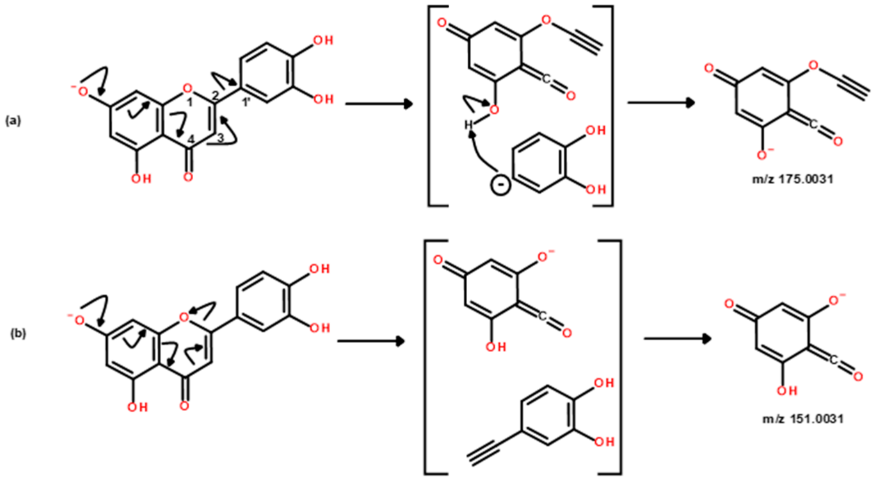

Figure S1. Proposed mechanisms to rationalize aglycon dissociations via stepwise competitive processes (see Figure 3 for more examples of stepwise processes) by cleavage of (a) C(2)-C(11) linkage cleavage and proton transfer to yield *m/z* 175 and (b) C(2)-O bond cleavage resulting in *m/z* 151.

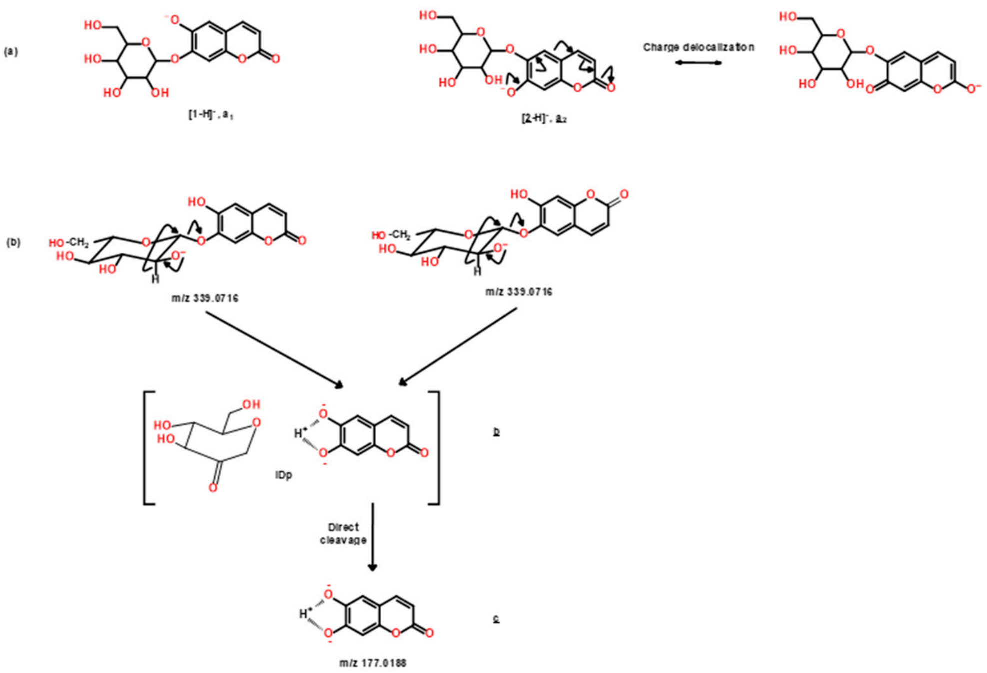

Figure S2. Comparison of the phenol stabilization by charge delocalization on deprotonated cichoriin [**1**-H]$^-$ and aesculin [**2**-H]$^-$, (b) stepwise dehydrated glucose release from particular [**1**-H]$^-$ and [**2**-H]$^-$ deprotomers through isomerization into a common ion/dipole complex (ID_P), induced by internal 1-2 hydride transfer from the CH-O$^-$ site of to the quinone aglycone moiety prior to dissociation. Splitting of the ID_P intermediate yields the deprotonated esculetin and dehydrated hexose neutral as final state (see more details in Figure 3).

C1 - Internal use

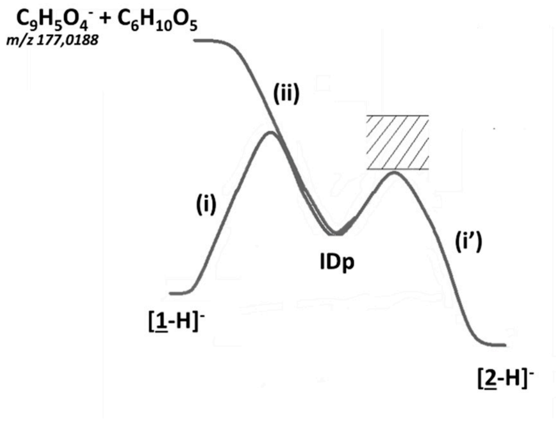

Figure S3. Qualitative energy pathways of the stepwise dissociation of both the cichoriin [1-H]⁻ and aesculin [2-H]⁻ ions through the endothermic (i) and (i') isomerization (see Figure 3) into a common ID_P intermediate prior to the (ii) dissociation into a common product ion *m/z* 177 by release of dehydrated hexose. Transition state position of the (i') pathway relative to that of (i) is difficult to be localized without quantum calculations.

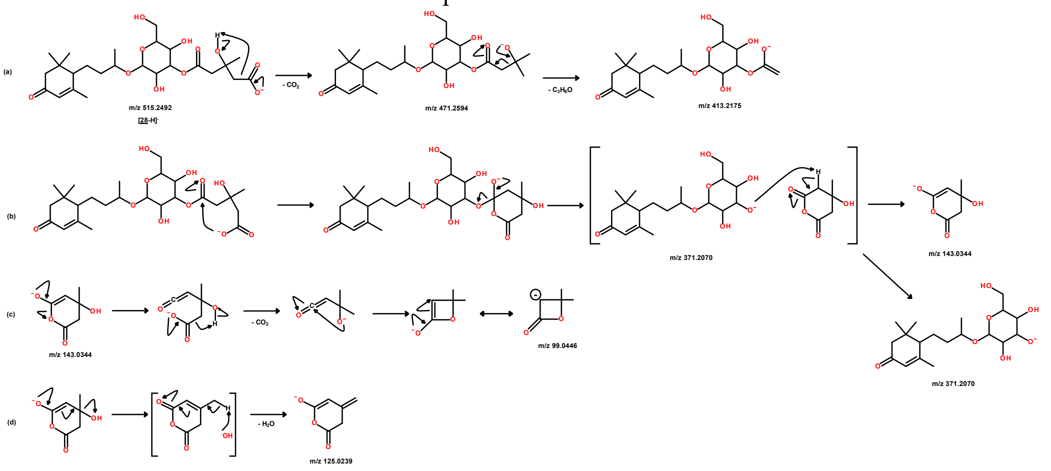

Figure S4. Proposed mechanisms of 3-hydroxy-3-methylglutaryl end group dissociations yielding (a) the consecutive losses of CO_2 and (CO_2 + C_3H_6O), (b) the complementary *m/z* 143 and *m/z* 371 ions via ion/dipole complex intermediate, and the *m/z* 143 competitive dissociations to form ions (c) *m/z* 99 and (d) *m/z* 125.

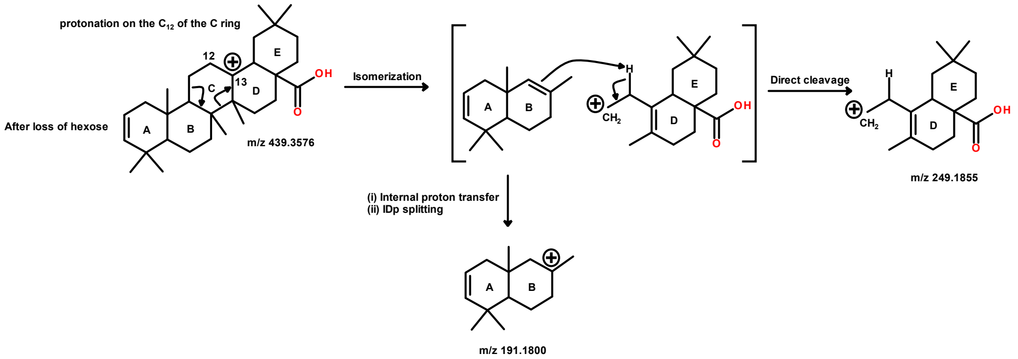

C1 - Internal use

Figure S5. Proposed stepwise dissociation mechanisms of the protonated molecule at the C(12)-C(13) double bond *via* ion/dipole complex intermediate consisting in both A/B and D/E unsaturated (and substituted) decalin systems followed either (i) by direct splitting (formation *m/z* 249.1848, $C_{16}H_{25}O_2^-$ stabilized by 1-2 hydride transfer yielding charge migration at allylic position and charge delocalization with the conjugate unsaturation) or (ii) by internal proton transfer followed by the ion/dipole splitting, yielding m/z 191.1792 product ion ($C_{14}H_{23}$ as tertiary carbonium).

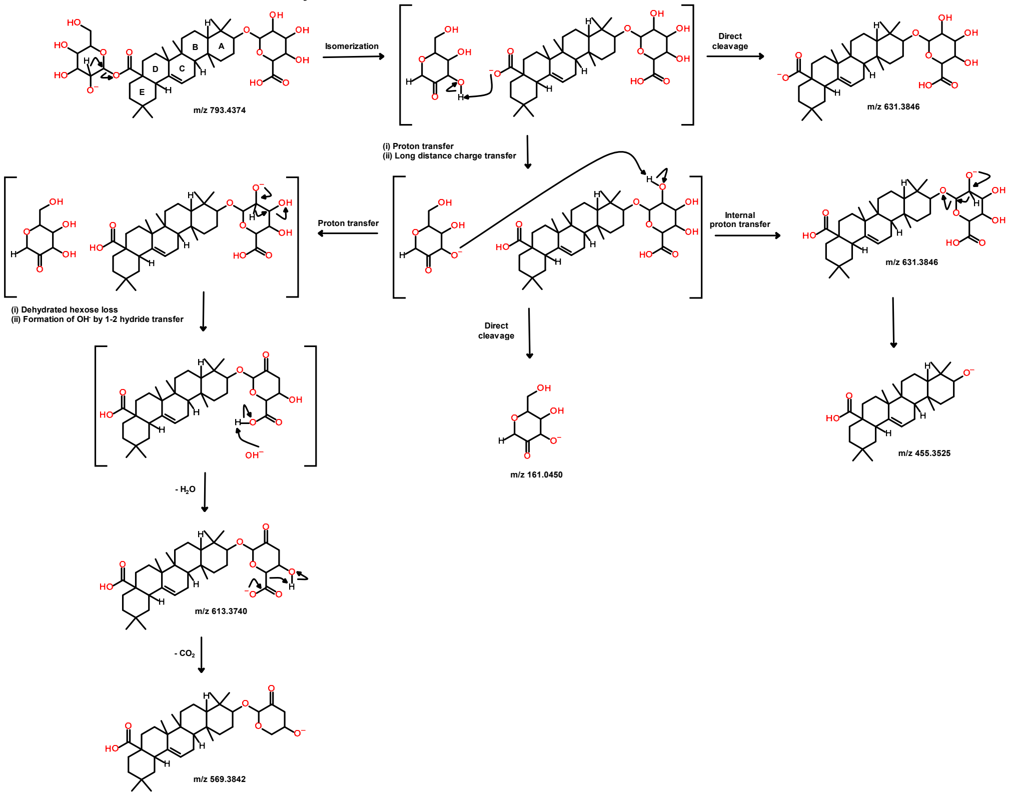

Figure S6. Proposed mechanisms of protonated calenduloside F dissociation initiated by the specific C-O ester bond cleavage at the D/E ring junction preferred to that of the O-C ether bond at the A ring of the aglycone. This requires stepwise processes based on the isomerization of the deprotonated molecule into an ion/dipole complex intermediate (as described in Figure 3) to form product ions: either by direct ID_P splitting (loss of dehydrated hexose) to give the *m/z* 631 ion, or after long-distance proton transfer from the D/E ring junction to the A ring, enabling release of dehydrated glucuronic acid by cleavage of the C-O ether linkage (*m/z* 455) in competition with formation of *m/z* 161. These losses compete with the release of H_2O and CO_2 to form *m/z 613* and *m/z 560*, respectively.

C1 - Internal use

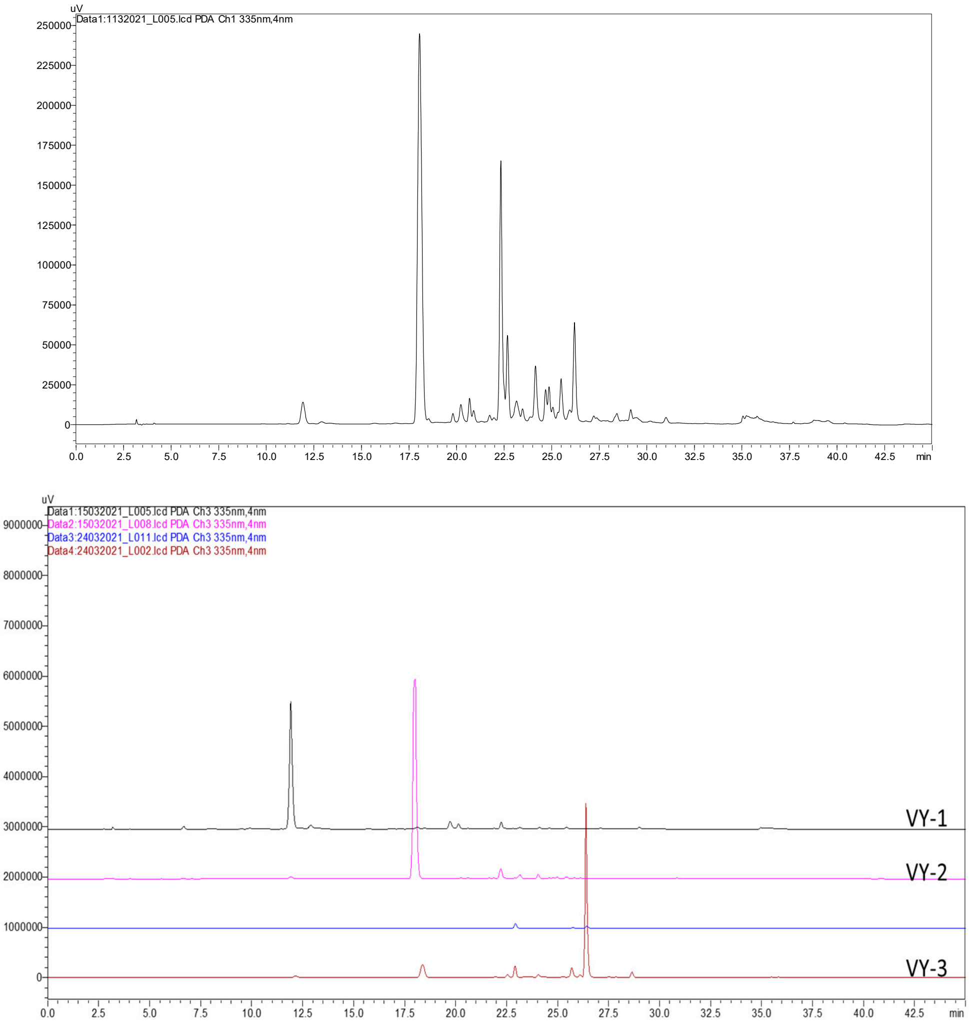

Figure S7. HPLC chromatogram of hydroalcoholic extract and fractions of *Viola yedoensis* by flash chromatography.

Table S1. Mobile phase gradient used in flash chromatography.

Time (mins)	% A (MeOH)	% B (Water)
0	100	0
5	95	5
10	90	10
15	85	15
20	80	20
50	50	50

C1 - Internal use

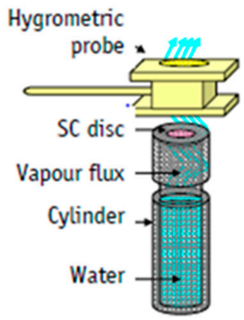

Figure S8. Hygrometric probe.

C1 - Internal use
